# Understanding immune protection against tuberculosis using RNA expression profiling

**DOI:** 10.1016/j.vaccine.2015.05.025

**Published:** 2015-09-29

**Authors:** Ulrich von Both, Myrsini Kaforou, Michael Levin, Sandra M. Newton

**Affiliations:** a Section of Paediatrics and Wellcome Trust Centre for Clinical Tropical Medicine, Division of Infectious Diseases, Department of Medicine, Imperial College London, United Kingdom; b Department of Genomics of Common Disease, School of Public Health, Imperial College London, London, United Kingdom

**Keywords:** TB, tuberculosis, MTB, *Mycobacterium tuberculosis*, IFN-γ, interferon-gamma, PBMC, peripheral blood mononuclear cells, MSMD, Mendelian susceptibility to mycobacterial disease, BCG, bacille Calmette–Guerin, LTBI, latent tuberculosis infection, Transcriptomics, RNA expression profiling, Tuberculosis, Vaccines, Interferon-γ, Type I interferon

## Abstract

A major limitation in the development and testing of new tuberculosis (TB) vaccines is the current inadequate understanding of the nature of the immune response required for protection against either infection with *Mycobacterium tuberculosis* (MTB) or progression to disease. Genome wide RNA expression analysis has provided a new tool with which to study the inflammatory and immunological response to mycobacteria. To explore how currently available transcriptomic data might be used to understand the basis of protective immunity to MTB, we analysed and reviewed published RNA expression studies to (1) identify a “susceptible” immune response in patients with acquired defects in the interferon gamma pathway; (2) identify the “failing” transcriptomic response in patients with TB as compared with latent TB infection (LTBI); and (3) identify elements of the “protective” response in healthy latently infected and healthy uninfected individuals.

## 1 Introduction

The development of improved vaccines against *Mycobacterium tuberculosis* (MTB) is a high priority for the global effort to reduce the incidence of tuberculosis (TB) [1]. Although bacille Calmette–Guerin (BCG) is one of the most widely used vaccines, and is administered soon after birth in many high burden countries, the vaccine has limited efficacy in preventing pulmonary TB in adults with efficacy ranging from 0 to 80% [2,3] and the duration of protection is unclear [4,5]. However BCG has largely been retained in childhood immunisation schedules because of its effect in reducing disseminated forms of the disease including TB meningitis [6,7]. In addition to the poor efficacy of the current vaccine, a major concern of its use in countries where TB and HIV are both epidemic, is the increasing risk of complications including lymphadenitis and disseminated BCG in immunosuppressed infants with HIV co-infection [8]. There is thus an urgent need for better vaccines; however current efforts have been hampered by our inadequate understanding of the basis of immune protection against TB [9,10].

A paradox in the search for biomarkers of protection against TB is the fact that all existing markers which detect an immune response to mycobacteria are unable to distinguish between a “successful” host immune response which results in long term immunity to TB, and the state of latency which may progress over time to active disease [11,12]. For example interferon-gamma (IFN-γ) response assays, which detect T cell reactivity to a range of mycobacterial antigens, are positive in both active disease and latent TB infection (LTBI) [13]. In the absence of a reliable marker of protective immunity, the selection of candidate vaccines or mycobacterial antigens for inclusion in new vaccines has relied on animal experiments which may not reflect immunity to MTB in man [14]. In the absence of biomarkers of protective immunity, large scale Phase III efficacy studies are the only means of evaluating new vaccines which are both costly and time consuming.

The introduction of genome wide RNA expression profiling has provided a powerful new method to interrogate the host response to mycobacteria [15–20]. In this article we review how RNA expression profiling might provide clues to the nature of protective immunity against MTB, and how it might be used be used to identify novel biomarkers of protective immunity that might be used in subsequent evaluation of novel vaccine candidates.

## 2 How to detect protective immunity to MTB

**Fig. 1:**
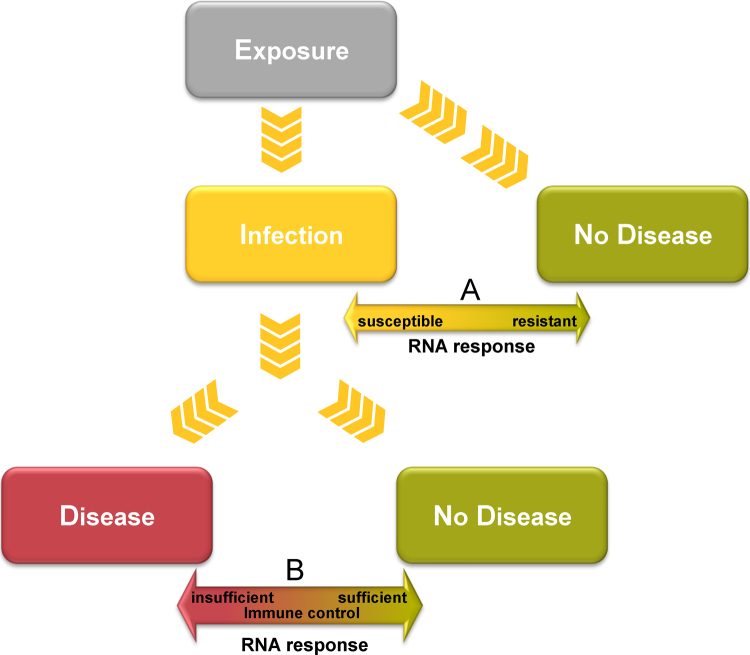
Potential comparisons through which RNA expression data might inform understanding of host responses to MTB infection. Comparison between infection and no disease (A) after exposure might reveal immune mechanisms underlying susceptibility and resistance. Comparison between disease and no disease after infection (B) might indicate pathways required to contain infection.

The growing number of published studies in which genome wide RNA expression profiling has been undertaken in the context of mycobacterial infection provides a vast amount of new data on the genomic response to TB [15,18,19,21,22]. However the major question is how to use this data to improve understanding of the basis of protective immunity. Ideally identification of a host RNA expression signature of the “protective” immune response would require prospective studies and long term follow up of individuals exposed to MTB, with comparison firstly of RNA expression in those individuals who remain uninfected (despite exposure) with those who develop disease (this would identify a disease susceptible/resistant response) and secondly comparison of those who once infected, remain with LTBI lifelong, with those who develop active disease (this would identify the immune response required to contain infection) (Fig. 1). However, in view of the low rate of progression to active disease and the relatively small numbers of those who after exposure develop TB disease, such studies require following up large numbers of patients for many years; nevertheless these are being undertaken. As an alternative approach, we propose that the nature of the protective transcriptomic response to mycobacteria might be identified by studying the following patient groups:

1. Patients with specific inherited or acquired immune defects that predispose to mycobacterial infection.
  The discovery of Mendelian single gene defects in the IFN-γ and IL-12 pathway that confer greatly increased risk of progressive mycobacterial disease, has implicated a number of immunological active molecules and pathways that appear to be essential for containment of mycobacteria [23,24]. The identification of acquired defects in the IFN-γ response pathway due to auto-antibodies against IFN-γ, similarly provides a clue to the immune pathways essential for successful containment of mycobacteria [25]. Studies of the transcriptomic differences between healthy individuals, and those with defects in the IFN-γ/IL-12 pathway might identify transcriptomic responses that are essential for protective immunity to MTB.
2. Comparison of the failed and successful immune response to mycobacteria.
  Patients with active TB can be considered as being unable to contain mycobacterial infection. In contrast, healthy individuals with LTBI are able to successfully contain mycobacterial infection at least for the time that they remain free of disease. Comparison of the transcriptome of healthy uninfected individuals, with that of active disease, or LTBI might provide clues to what constitutes a protective host response to TB.
3. Comparison of highly exposed individuals who remain life long without evidence of LTBI, those with long-term latency who do not progress to disease and those with LTBI who develop active disease.
  These three groups of patients may be highly informative as to the nature of the protective immune response and the specific transcriptomic response that distinguishes resistance to infection, and resistance to development of disease either short or long term.

By studying these patient groups we will illustrate the potential of the recently available transcriptomic data to be applied to addressing these questions.

## 3 Identifying transcriptomic responses in patients with inherited or acquired mycobacterial susceptibility defects

Mendelian susceptibility to mycobacterial disease (MSMD) is an inherited disorder characterised by predisposition to clinical disease caused by weakly virulent mycobacteria such as poorly pathogenic strains (non-tuberculous mycobacteria) and *Mycobacterium bovis* BCG (vaccine strains) [26]. Patients are also more susceptible to salmonellosis, candidiasis and TB [24]. In 1996, our group and that of Casanova reported mutations in the gene encoding the IFN-γ receptor, leading to absence of the IFN-γ receptor 1 chain as a cause of mycobacterial susceptibility [27,28]. Mutations in seven autosomal (IFNGR1, IFNGR2, IL12B, IL12RB1, STAT1, IRF8, ISG15) and two X-linked (NEMO, CYBB) genes have since been identified in patients with MSDM [29–33], which all result in impaired IFN-γ mediated immunity to infection [23]. However those patients with defects particularly in the IFNGR1 [34] and IL-12p40 [35] are found to be particularly susceptible to mycobacterial infection, including MTB [23]. These reports collectively demonstrate that up-regulation of macrophage mycobactericidal mechanisms through the IFN-γ/IL-12 pathway is critical for a successful immune response to mycobacteria.

In 2005, we reported 3 patients with severe, unexplained, non-tuberculous mycobacterial infection [25]. We isolated an anti-IFN-γ antibody from the patients’ plasma and showed that the antibody was functional in (a) blocking the up-regulation of TNF-alpha production in response to endotoxin, (b) inhibiting transcription of the IFN-γ inducible genes and (c) inhibiting up-regulation of HLA class II expression on peripheral blood mononuclear cells (PBMCs). This report highlighted that predisposition to mycobacterial susceptibility can be a result of acquired defects in macrophage activation by IFN-γ, in addition to genetic defects in the IFN-γ/IL-12 pathway [25], and many other patients with acquired IFN-γ pathway defects due to auto-antibodies have now been reported [36–38].

**Fig. 2:**
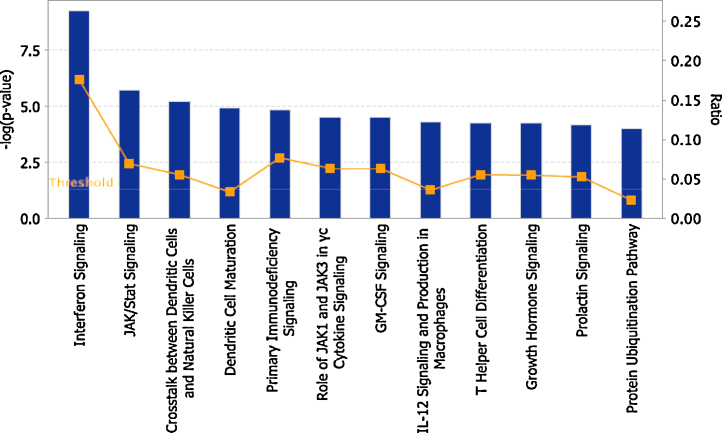
Biological pathways impaired in the presence of purified anti-IFN-γ antibody from a patient with acquired susceptibility to TB. 64 genes, under expressed in the presence of an anti-IFN-γ antibody [25], were assigned to biological pathways using Ingenuity pathway analysis (IPA^®^). Only genes showing 2.5 fold difference between control (no antibody) and treated cells (anti-IFN-γ antibody) were considered to be under-expressed. Top 12 pathways are shown and ranked by significance. Only entities that have a −log (*p*-value) of greater than 4 are displayed. Orange points connected by a thin line represent the ratio of significantly differentially expressed genes in each pathway divided by the total number of genes that make up that entire pathway. (For interpretation of the references to colour in this figure legend, the reader is referred to the web version of this article.)

To further explore the immunological consequences of impairment of the IFN-γ response pathway by anti-IFN-γ antibodies, we analysed the transcriptomic response of PBMCs from a healthy donor stimulated with IFN-γ in the presence or absence of the purified IFN-γ antibody [25]. Sixty four genes were found to be significantly differentially under-expressed in the presence of the purified IFN-γ antibody [25], and these might be considered potential biomarkers of the “susceptible” interferon response, and indicative of the key biological pathways required for protection against mycobacteria. When these genes were assigned to biological pathways using Ingenuity pathway analysis software, we found impairment of multiple pathways in addition to those known to be involved in interferon signalling, including pathways such as JAK/STAT signalling, crosstalk between dendritic cells and NK cells, GM-CSF signalling, IL-12 signalling and production in macrophages or T-helper cell differentiation (Fig. 2).

The genes and pathways showing impaired induction in the presence of anti-IFN-γ antibodies may provide clues as to what distinguishes the protective and susceptible genomic response to mycobacterial infection. Such comparisons between existing and new transcriptomic datasets may thus yield insight into the genes and biological pathways that are essential for protection against mycobacteria.

## 4 Type I and type II interferon balance – a key to protection against TB?

Identification of how the “failed” transcriptomic response in patients with active TB, differs from the “successful” response in healthy individuals with LTBI, might provide important clues to the nature of the protective immune response. We focused on type I (α, β) and type II (γ) IFNs that have increasingly been implicated in mycobacterial immunity.

Although both types of interferon are important in activating the host immune response, IFN-γ is a potent activator of macrophage mycobactericidal mechanisms, and its essential role in protective immunity to mycobacteria has been definitively established by the identification of the human gene defects in the IFN-γ pathway as discussed above. In contrast, the role of the type I IFNs in facilitating mycobacterial growth has been recognised recently. In several studies in both mice and humans, overproduction of type I IFNs has been associated with exacerbation of TB [15,19,39], and the transcriptomic signature of active TB has been reported to be characterised by an excess of type I IFNs [19,21,39]. Ottenhoff et al. [15] elucidated a predominant type I IFN mediated transcriptional signature in patients with active TB disease that normalised during treatment coincident with up-regulation of IFN-γ. As a consequence of these findings, type I IFNs and their signalling cascade have been proposed as potential quantitative tools for monitoring active TB [15]. Type I IFNs act to increase production of immune-suppressive IL-10 and block the responsiveness to IFN-γ and, as a consequence, IL-12 production in an IL-27 independent manner [39]. IL-10 plays a key role in this regulatory system. Suppression of TNFα- and IL-1β-production has also been attributed to type I IFNs.

Mayer-Barber et al. have recently suggested a complex interplay of interleukin-1 (IL-1), type I IFNs and eicosanoids in controlling intracellular growth of MTB [40]. They showed that IL-1 confers host resistance to MTB through the induction of eicosanoids that limit excessive type I IFN production and reduce growth of MTB. Reduced IL-1 responses and/or excessive type I IFN induction were shown to be linked to an eicosanoid imbalance associated with disease exacerbation. In LTBI there appears to be a balance between IL-1 and type I IFNs, in part mediated by prostaglandin E2, which prevents uncontrolled inflammation. If this balance is disturbed (as appears to be the case in patients with TB), unopposed activity of type I IFNs occurs which further suppresses the secretion of IL-1α and IL-1β mediated by the activity of IL-10 and IL-1 receptor antagonist [41]. This in turn blocks the production of PGE2 and the enzyme cyclooxygenase-2 (COX-2). This data suggests that the inflammatory cycle driven by type I IFNs is enhanced in active TB, promoting mycobacterial growth.

However, the model in which type I IFNs are seen as “bad” and type II IFNs “good” for containment of MTB, has largely been based on murine and cellular experiments, and may not reflect the situation in human TB. The availability of RNA transcriptome data from patients with active TB and LTBI provides a new way of exploring the balance between type I IFNs and IFN-γ in patients with active TB disease. In order to establish the relative expression of genes that are controlled by different IFN types in TB patients, we identified the genes that are up- or down-regulated in response to stimulation of PBMCs with specific IFNs using the data of Waddell et al. [18]. In this study genes that are up-regulated or down-regulated in healthy individuals upon *in vitro* stimulation of PBMCs with IFN-α, IFN-β and IFN-γ are identified and reported. We then explored the overlap of IFN-α, IFN-β and IFN-γ inducible genes amongst all the genes that were significantly differentially expressed (SDE) between TB patients and healthy individuals with LTBI (HIV uninfected) using our published RNA expression data [42].

**Fig. 3:**
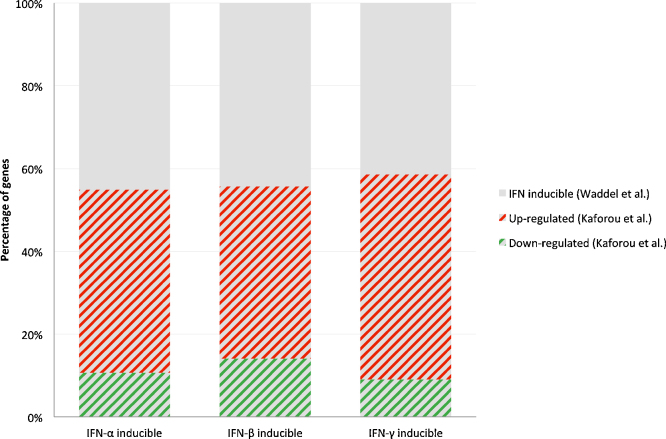
Percentages of up or down regulated genes, that are differentially expressed in TB *versus* LTBI, amongst IFN (α, β, γ) inducible genes. In the study of Waddell et al. [18], 226, 370 and 111 genes were identified by microarray analysis as being induced in PBMCs stimulated with IFN-α, IFN-β and IFN-γ respectively. Of these, 225, 367 and 111 genes (IFN-α, IFN-β and IFN-γ respectively) were present in the microarrays used in the study by Kaforou et al. [42]; 124 (55.1%), 206 (56.3%) and 65 (58.6%) of these were detected as being significantly differentially expressed (adjusted *p* value < 0.05) between TB patients and LTBI individuals with 80.7%, 74.7% and 84.6% classified as up-regulated and 19.3%, 25.3%, 15.4% as down regulated.

As shown in Fig. 3, patients with active TB show up-regulation of a high proportion of the genes induced by both type I IFNs and IFN-γ. A surprising finding from this analysis is that patients with active TB show up regulation of both type I IFN and IFN-γ induced genes; we did not observe an “imbalance” between genes induced by type I IFNs or IFN-γ (excessive type I IFN production and reduced IFN-γ production) as we may have expected. Our analysis suggests that active TB occurs in the presence of a robust IFN-γ response, as well as an active type I IFN response, and does not support the simplistic view of disease occurring in the context of excess type I IFNs and defective IFN-γ responses. A major question for future studies is why an apparently robust IFN-γ response is ineffectual in containing the infection.

## 5 Identifying “protective” transcriptomic responses: is IL-32 a biomarker of the protective immune response?

In a novel approach to identifying “protective” transcriptional responses to TB, a recent publication linked both *in vitro* data of the transcriptional response in macrophages and published *in vivo* transcriptome studies [43]. The authors studied RNA expression in human macrophages infected with MTB and used network analysis of the expressed genes to identify a set of interconnected genes that corresponded with a network of IL-15 induced defence-response genes. The hub gene in this network was IL-32 which was shown to be adequate alone to induce expression of antimicrobial peptides such as cathelicidin and DEFB4, at levels comparable with stimulation by IFN-γ or IL-15. They also showed that IL-32 is a functional mediator of IFN-γ induced human macrophage mycobactericidal activity.

In order to identify molecular markers of host defence, Montoya et al. examined the overlap between the gene set induced by IL-15 and IL-32 *in vitro*, and those expressed *in vivo* [43]. The authors hypothesised that genes up-regulated in both LTBI and healthy uninfected individuals are involved in maintaining MTB in a dormant state and prevent transition to active disease. They employed pairwise comparisons of gene expression in five published clinical data sets [19,42,44,45] of LTBI *versus* active TB, two data sets comparing LTBI *versus* healthy controls [19,44] and one dataset including patients with active TB undergoing chemotherapy [45]. This analysis identified eight genes (also members of the IL-15 induced gene set) that were significantly up-regulated in LTBI *versus* active TB and LTBI *versus* healthy controls, with varying degrees of agreement between datasets. However, IL-32 was the only gene up-regulated in all data sets. Based on this analysis, it was concluded that IL-32 might serve as a molecular marker of LTBI and may be a mediator of the “protective” immune response that successfully contains MTB in the latent state. This exciting analysis demonstrates the power of RNA transcriptomic analysis to reveal new biological insights, and warrants confirmation in prospective studies.

## 6 Conclusions

We have presented a number of examples of how published genome wide RNA expression data, derived from microarray studies of both *in vitro* and *in vivo* mycobacterial infection can be used to identify and characterise the “protective” immune response. Although we have focused on IFN induced genes, and the recently identified IL-32 pathway, these are only initial examples of how transcriptomic studies can be interrogated to understand the basis of protection against MTB and other mycobacteria. Future studies linking carefully phenotyped patient groups, monitored over time, with transcriptomic and genetic analysis, are likely to help reveal the basis of immune protection against TB and thus make selection and evaluation of new vaccines possible on a more rational basis. While RNA expression can provide clues to the overall pattern of gene activation or repression in different clinical responses to TB, proof that any given pattern of RNA expression will be “protective” if induced by a candidate vaccine, will ultimately require verification in future clinical studies.
